# Extensive Cerebral Venous Sinus Thrombosis at High Altitude

**DOI:** 10.7759/cureus.85742

**Published:** 2025-06-10

**Authors:** Jamal Ouachaou, Driouich Aicha, Mohammed Sidayne, Fatimazahrae El Khettab, Youssef Zarrouki

**Affiliations:** 1 Anesthesiology and Intensive Care, Mohammed VI University Hospital of Marrakech, Faculty of Medicine and Pharmacy, Cadi Ayyad University, Marrakech, MAR

**Keywords:** acute mountain sickness, anticoagulation, cerebral edema, cerebral venous sinus thrombosis, cerebrovascular disease, high altitude, magnetic resonance imaging (mri)

## Abstract

Rapid ascent to altitudes above 8,000 feet (2,500 meters) is known to be associated with acute mountain sickness (AMS), but its role as a cause of cerebrovascular disorders is rarely described. Cerebral venous sinus thrombosis is a rare and potentially fatal condition that can frequently be misdiagnosed.

In this article, we report an unusual case of a 27-year-old woman who developed an extensive cerebral venous sinus thrombosis (CVST) during an expedition in the Atlas Mountains. The underlying mechanisms explaining this event will be discussed in light of this clinical observation.

## Introduction

Cerebral venous sinus thrombosis (CVST) is an uncommon cause of cerebral edema and infarction [1]. Diagnosis can be challenging due to the lack of specific symptoms. However, it is important to consider, as it is associated with significant morbidity and mortality. Exposure to high altitude presents various challenges (hypoxia, dehydration), which can lead to hypercoagulability. CVST is rarely described in the context of high altitude. We report the case of a young woman who developed extensive CVST during a mountain expedition. Her outcome was favorable due to early diagnosis and timely treatment.

## Case presentation

A healthy 27-year-old woman, a non-smoker and not using hormonal contraceptives, experienced severe headaches on the sixth day of her climb in the Atlas Mountains (13,671 feet (4167 meters), the highest peak in North Africa). These headaches were accompanied by persistent vomiting, which did not improve even after descending to a lower altitude. At a local emergency room, physical examination showed normal mental status with no neurological deficits. She was tachycardic (122 bpm), normotensive, and slightly tachypneic, with signs of dehydration. Laboratory analysis revealed a hematocrit (Hct) level of 47%. Fluid loading (2L) was started and continued during her transfer to our hospital.

Upon arrival, despite continued headaches and vomiting, her neurological examination showed no significant abnormalities. Hemodynamically, her heart rate decreased to 105 bpm, and her hydration status improved. Follow-up lab tests showed a decrease in hemoconcentration, hemoglobin 15g/dl (Hct 40%), urea 0.52 g/l, creatinine 90,3 umol/L, sodium 139 mmol/l, and potassium 3.6 mmol/l (Table 1). 

**Table 1:** Laboratory findings.

Test	Result (SI Units)	Normal Range (SI Units)
Hemoglobin G/dl	9.93	8.4 – 11.2
Hematocrit %	0.40	0.40 – 0.54
White Blood Cells G/L	13	4 – 10
Platelets G/L	250	150 – 400
Urea (mg/L)	0,52	0,25-0,48
Creatinine µmol/L	90,3	60 – 110
Sodium mmol/L	139	135 – 145
Potassium mmol/L	3.8	3.5 – 5.0
Prothrombin Ratio%	96	70 – 100

**Figure 1:**
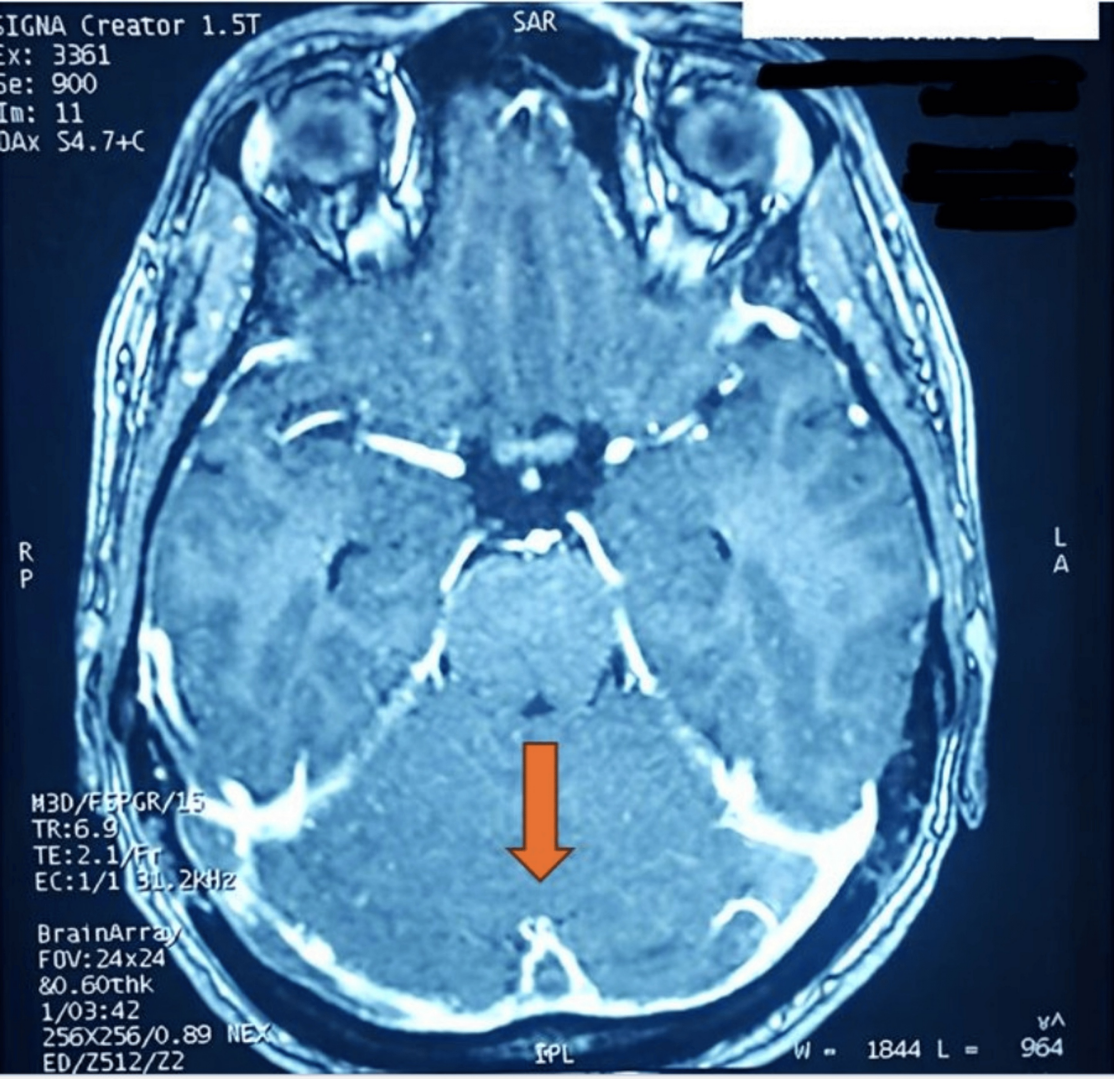
Thrombosis of superior sagittal and transverse sinuses with the empty delta sign (orange arrow)

A brain magnetic resonance imaging (MRI) revealed thrombosis of the superior sagittal sinus with a characteristic empty delta sign and extensive thrombosis of the transverse sinus, extending to the right internal jugular vein (Figure 1). 

Once the CVST diagnosis was confirmed, and in light of the signs of increased intracranial pressure, the patient received a single dose of mannitol (1 g/kg) and anticoagulation with enoxaparin. Antiepileptic therapy and symptomatic treatment for headache and vomiting were also administered. Laboratory screening for thrombophilia (lupus anticoagulant, anticardiolipin antibodies, Factor V Leiden mutation, deficiencies of antithrombin, protein C, or protein S) revealed no abnormalities. The clinical course was favorable, and the patient did not develop any neurological deficits. She was discharged on day nine with a prescription of valproic acid and adjusted vitamin K antagonist therapy to maintain an international normalized ratio (INR) of 2.5.

## Discussion

CVST is a rare condition, affecting three to four people per million [2], and is defined by the formation of blood clots in the cerebral veins or dural sinuses. Over the past few years, its diagnosis has become more common due to increased awareness and the widespread availability of MRI [3]. Risk factors for CVST include infection, neoplasm, pregnancy, the postpartum period, systemic diseases, oral contraceptive use combined with tobacco smoking, and intrinsic coagulopathies. The patient in this case had no obvious risk factors, except for high-altitude exposure.

When neurological signs persist even after descending, MRI should be performed, as the symptoms are unlikely to be solely due to acute mountain sickness (AMS). AMS is a neurological syndrome characterized by headache, anorexia, nausea, vomiting, insomnia, and fatigue [4]. It results from brain swelling and intracranial hypertension, which can lead, in extreme cases, to high-altitude cerebral edema (HACE). AMS is believed to be caused by local vasodilation of cerebral blood vessels in response to hypoxia, leading to increased blood flow, capillary pressure, and leakage [5].

The presumed mechanisms of CVST in our patient likely involved a combination of factors: dehydration leading to relative polycythemia, altitude acclimatization polycythemia as a response to hypoxemia, the increased oxygen demand from physical exertion during the climb, and raised intracranial pressure from AMS, which slowed venous blood flow due to extrinsic compression. Treatment for CVST typically involves low-molecular-weight heparin during the acute phase, antiepileptic treatment for patients with seizures or supratentorial lesions, and decompressive surgery in severe cases to prevent brain herniation [3].

Prevention of CVST at high altitude is crucial, particularly given the pathophysiological overlap with AMS. Mountaineers should regulate their rate of ascent to minimize the risk. Above 2,500 m, it is recommended to avoid ascending more than 300 to 500 m per day. Additionally, climbers should take a rest day every 600 to 900 m and maintain adequate hydration [6,7].

## Conclusions

CVST should be considered in mountaineers who present neurological deterioration that cannot be solely attributed to AMS. MRI should be performed if neurological signs persist despite descending. Increased awareness among climbers, particularly in mountain tourism areas, is essential to reduce the occurrence of this severe condition.
